# JTT-130, a Novel Intestine-Specific Inhibitor of Microsomal Triglyceride Transfer Protein, Reduces Food Preference for Fat

**DOI:** 10.1155/2014/583752

**Published:** 2014-05-15

**Authors:** Yasuko Mera, Takahiro Hata, Yukihito Ishii, Daisuke Tomimoto, Takashi Kawai, Takeshi Ohta, Makoto Kakutani

**Affiliations:** Central Pharmaceutical Research Institute, Japan Tobacco Inc., 1-1 Murasaki-cho, Takatsuki, Osaka 569-1125, Japan

## Abstract

Microsomal triglyceride transfer protein (MTP) is involved in the assembly and secretion of triglyceride-rich lipoproteins from enterocytes and hepatocytes. JTT-130 is a novel intestine-specific MTP inhibitor, which has been shown to be useful in the prevention and treatment of dyslipidemia, obesity, and diabetes. JTT-130 has also been shown to suppress food intake in a dietary fat-dependent manner in rats. However, whether JTT-130 enables changes in food preference and nutrient consumption remains to be determined. Therefore, the aim of the present study was to investigate the effects of JTT-130 on food preference in rat under free access to two different diets containing 3.3% fat (low-fat diet, LF diet) and 35% fat (high-fat diet, HF diet). JTT-130 decreased HF diet intake and increased LF diet intake, resulting in a change in ratio of caloric intake from LF and HF diets to total caloric intake. In addition, macronutrient analysis revealed that JTT-130 did not affect carbohydrate consumption but significantly decreased fat consumption (*P* < 0.01). These findings suggest that JTT-130 not only inhibits fat absorption, but also suppresses food intake and specifically reduces food preference for fat. Therefore, JTT-130 is expected to provide a new option for the prevention and treatment of obesity and obesity-related metabolic disorders.

## 1. Introduction

Western diets that consist of high levels of fat have been closely related to dyslipidemia, obesity, and the induction of insulin resistance [1–3]. Fat contains more energy per unit weight than carbohydrates or proteins and, as a result, high-fat diets bring in more energy than low-fat diets at the same unit weight, leading to obesity. In addition, diets with a higher fat energy ratio are more likely to induce obesity even if the total daily energy is the same [4]. There have been reports that obese individuals have a higher preference for consuming fat than non-obese individuals [5–7]. Therefore, it may be important to decrease fat consumption and to correct the food preference for fat in the prevention and treatment of obesity and obesity-related metabolic disorders.

Microsomal triglyceride transfer protein (MTP) plays a pivotal role in the mobilization and secretion of triglyceride-rich chylomicrons in the enterocytes and very low-density lipoproteins (VLDL) in hepatocytes [8–10]. In particular, intestinal MTP plays a critical role in the absorption of dietary lipids, such as fat and cholesterol [11]. JTT-130, [diethyl-2-({3-dimethylcarbamoyl-4-[(4′-trifluoromethylbiphenyl-2-carbonyl)amino]phenyl }acetyloxymethyl)-2-phenylmalonate], a novel intestine-specific MTP inhibitor, was designed to be rapidly hydrolyzed and inactivated by the cleavage of the ester group in the structure immediately after intestinal absorption in order to avoid inhibition of hepatic MTP resulting in hepatic steatosis [12]. In our previous reports, we showed that JTT-130 may be useful in the prevention and treatment of dyslipidemia [12], obesity [13], and type II diabetes [14] in animals. In particular, JTT-130 showed food suppressive effect in a dietary fat-dependent manner in rats fed with diets differing in fat content [15]. As the mechanism of action, we already demonstrated that this effect may be attributed to free fatty acids that have accumulated in the gastrointestinal tract as a result of inhibition of fat absorption. Recently, the gastrointestinal tract, which is the largest endocrine organ in the body, has been observed as playing important roles in the regulation of energy homeostasis, as well as maintaining its primary function in the digestion and absorption of nutrients [16–18]. Therefore, we postulated that JTT-130 may change food preference and nutrient consumption, in addition to reducing food intake. However, there has been no information to date about the effect of other MTP inhibitors on food preference. In this study, to test our hypothesis, we administered JTT-130 to rats with free access to two diets, one containing 3.3% and the other 35% fat, and determined each diet intake every day to analyze macronutrient consumption.

## 2. Materials and Methods

### 2.1. Chemicals

JTT-130, diethyl-2-({3-dimethylcarbamoyl-4-[(4′-trifluoromethylbiphenyl-2-carbonyl) amino]phenyl} acetyloxymethyl)-2-phenylmalonate, was synthesized by Japan Tobacco Inc. (Osaka, Japan). All other reagents used in this study were obtained commercially.

### 2.2. Animals and Diets

Male Sprague-Dawley rats (six weeks) were obtained from Charles River Japan Inc. (Yokohama, Japan) and maintained at a room temperature of 23 ± 3°C and an air humidity of 55 ± 15% in a 12-/12-hour light/dark cycle (lights on at 8:00 AM; lights off at 8:00 PM). Animals were given free access to water and experimental diets (Table 1). The diets were a 3.1% fat diet, a 3.3% fat diet (LF diet) and a 35% fat diet (HF diet) obtained from Oriental Yeast Co., Ltd. (Tokyo, Japan) or Research Diets Inc. (New Brunswick, NJ). After acclimation under these conditions over 2 weeks, the rats were randomized into the control or JTT-130 treatment group by matching food intake, total caloric intake, ratio of HF diet to total caloric intake, body weight, and levels of body weight gain. All procedures were conducted according to the Japan Tobacco Animal Care Committee's guidelines.

### 2.3. Evaluation of Food Intake and Body Weight

Rats were individually housed and given free access to LF and HF diet, and the following experiment was conducted. The rats were given vehicle (0.5% methylcellulose solution) or JTT-130 at a dose of 10 mg/kg orally once daily (before the start of the dark cycle) for seven days, followed by a one-week recovery period. Before and during the treatment period and during the recovery period, LF and HF diet intake were determined daily before the start of the dark cycle. In addition, body weight was measured during the treatment period, and body weight gain was calculated.

### 2.4. Analysis of Caloric Intake and Macronutrient Consumption

Before and during the treatment period and during the recovery period, total caloric intake was calculated from the intake of two different diets and the respective caloric contents per unit weight (Table 1). The ratio of LF and HF diet intake was calculated as the ratio of caloric intake from each diet to total caloric intake. In addition, consumption of fat, carbohydrate, and protein was calculated from each diet and the content ratio of each component.

### 2.5. Statistical Analysis

Data are presented as means ± S.E. Statistical analysis was performed using SAS systems, version 8.2 (SAS Institute, Cary, NC). If equality of variances was indicated by an *F*-test, statistical analysis was performed using Student's *t*-test. If equality of variances was not indicated by an *F*-test, statistical analysis was performed using Welch's *t*-test. A *P* value <0.05 was considered statistically significant.

## 3. Results and Discussion

### 3.1. Effects of JTT-130 on Food and Caloric Intake from LF and HF Diets

**Figure 1:**
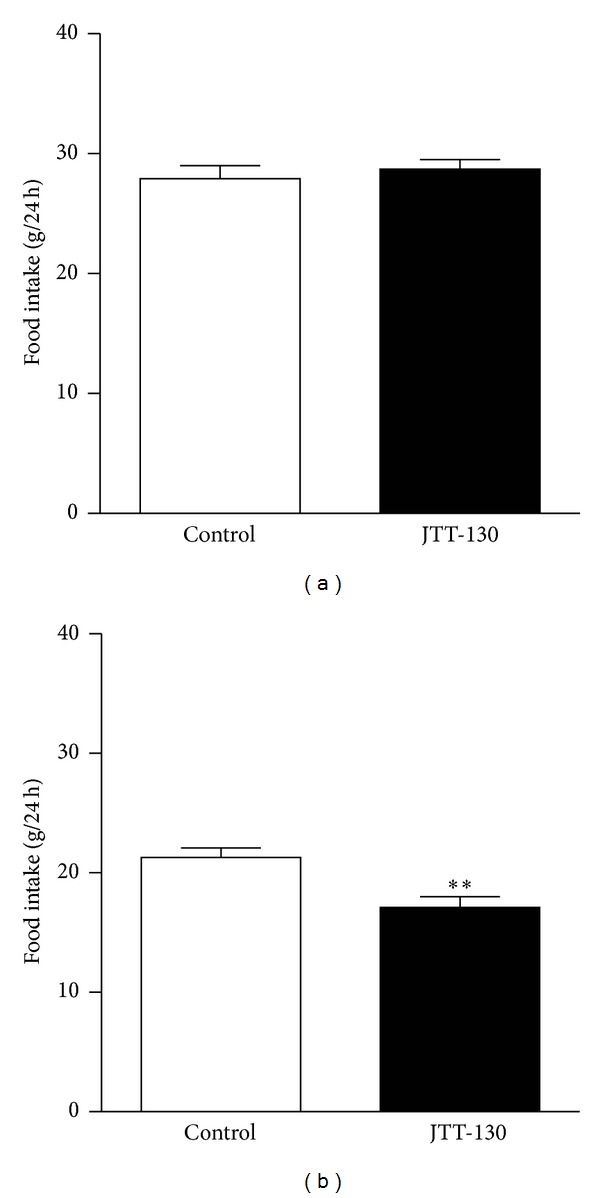
Effects of dietary fat on the suppression of food intake by JTT-130. Effect of JTT-130 on the suppression of food intake was assessed as described previously [15]. JTT-130 was administered orally to rats at a dosage of 10 mg/kg after 24 h food deprivation. Rats were allowed to have free access to 3.1% fat (a) or 35% fat (b) diets immediately after JTT-130 dosing. Cumulative food intake was monitored for up to 24 h after dosing JTT-130. Data are presented as means ± S.E. from six animals. **P < 0.01 versus control group.

As shown in Figure 1, treatment with JTT-130, which is an intestine-specific MTP inhibitor, led to a reduction in food intake in rats fed a 35% (w/w) fat diet, but not in rats fed a 3.1% (w/w) fat diet. This effect was consistent with the previous finding in which JTT-130 suppressed food intake in a dietary fat-dependent manner [15]. In addition, the major metabolite of JTT-130 did not reduce the food intake at doses up to 30 mg/kg in rats fed a 35% (w/w) fat diet (data not shown).

**Figure 2:**
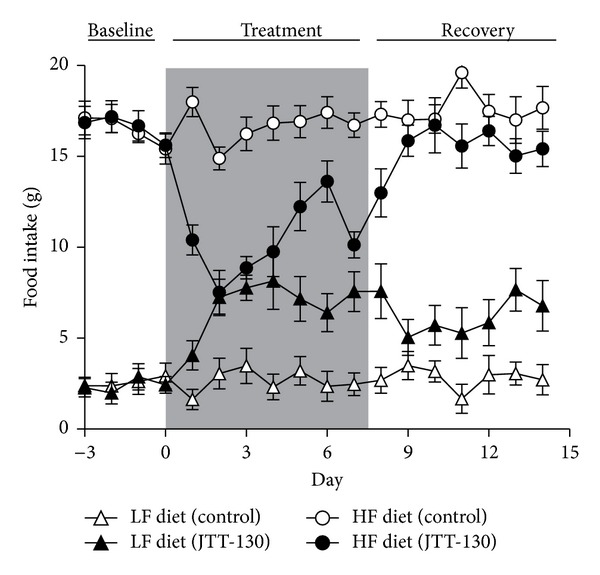
Effect of JTT-130 on LF and HF diet selection during the study period. Rats were allowed to have free access to LF and HF diet. JTT-130 was administered orally to rats at a dosage of 10 mg/kg for seven days. Data are presented as means ± S.E. from ten animals. Open circle: HF diet intake in the control group; closed circle: HF diet intake in the JTT-130 group; open triangle: LF diet intake in control group; closed triangle: LF diet intake in the JTT-130 group.

**Figure 3:**
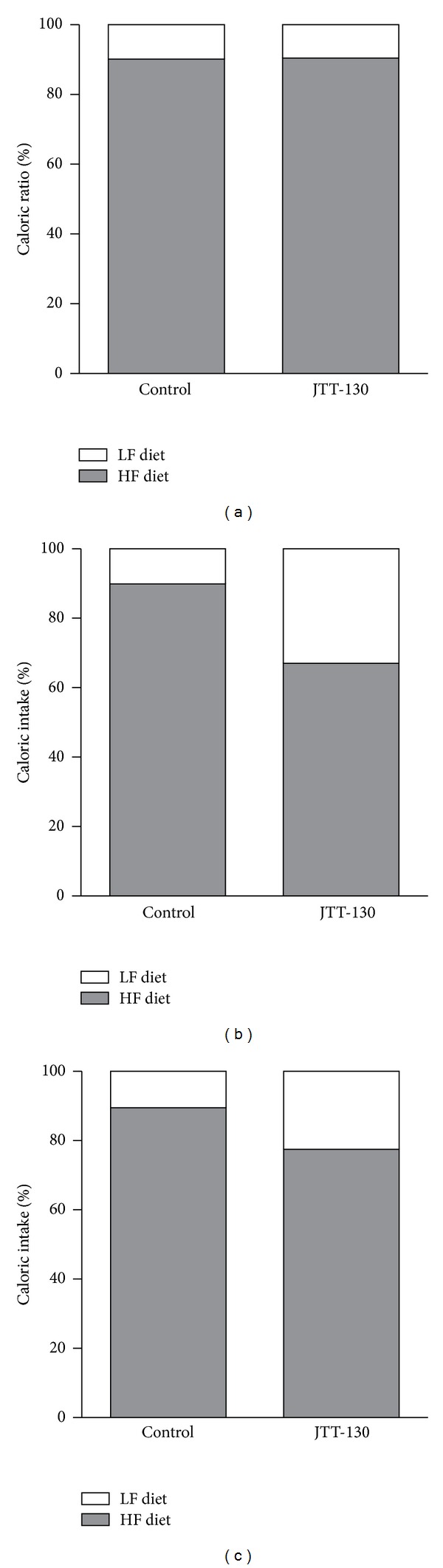
Effects of JTT-130 on caloric intake ratio from LF and HF diets. Caloric intake ratios were calculated from average daily intake from LF and HF fat dietsduring baseline (a), the treatment period (b), and recovery period (c). Data are presented as means ± S.E. from ten animals.

To investigate the effect of JTT-130 on food preference, we administered JTT-130 to rats with free access to two diets containing 3.3% (w/w) fat and 35% (w/w) fat (LF and HF diet) (Table 1) and determined LF and HF diet intake every day to analyze macronutrient consumption. Before treatment with JTT-130, rats preferred the HF diet to the LF diet. A greater daily caloric intake from the HF diet was observed in both the control (90.4%) and JTT-130 (90.2%) groups, with no differences observed between the two groups (Table 2 and Figures 2 and 3(a)). This higher preference for the HF diet in our experiment was consistent with the previous report wherein rats were shown to have a higher preference for fatty diets [19, 20].

**Figure 4:**
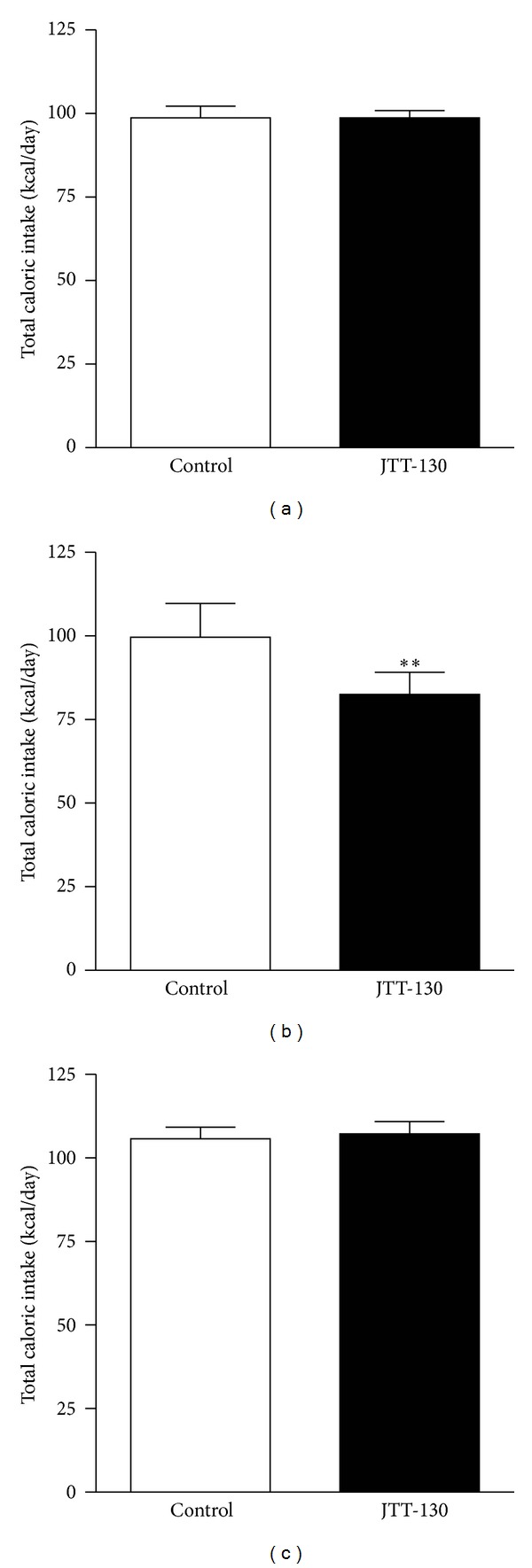
Effects of JTT-130 on total caloric intake. Total caloric intake was calculated from average daily intake of LF and HF diets during baseline (a), the treatment period (b), and recovery period (c). Data are presented as means ± S.E. from ten animals. **P < 0.01 versus control group.

During the seven-day treatment with JTT-130, there were no apparent changes from baseline in food intake in the control group, with an intake ratio of 10.0 ± 2.3% for the LF diet and 90.0 ± 2.3% for the HF diet (Table 2 and Figures 2 and 3(b)). In the JTT-130 group, food intake was characterized by decreased HF diet intake and increased LF diet intake compared with the control group (Table 2 and Figures 2 and 3(b)), with an intake ratio of 32.9 ± 3.8% for the LF diet and 67.1 ± 3.8% for the HF diet, showing that JTT-130 decreased the HF diet intake ratio to total caloric intake (Figure 3(b)). In addition, total caloric intake during this period was 100.3 ± 3.4 kcal/day in the control group and 82.3 ± 2.1 kcal/day in the JTT-130 group, which significantly decreased after treatment with JTT-130 (*P* < 0.01) (Figure 4(b)).

### 3.2. Effects of JTT-130 on Macronutrient Consumption

Since the results showed that JTT-130 changed the intake ratio of LF and HF diets, nutrient consumption was analyzed according to the following nutrient components: carbohydrates, proteins, and fats (Table 1). Fat consumption was 5.9 ± 0.2 g/day in the control group and 3.9 ± 0.2 g/day in the JTT-130 group, showing that JTT-130 significantly decreased fat consumption (Table 3). On the other hand, JTT-130 had no effect on carbohydrate consumption and slightly decreased protein consumption (Table 3). Since protein content (w/w (%)) was the same in both LF and HF diets, the decrease in protein consumption was presumably caused by a decrease in total food intake after treatment with JTT-130 and not by direct alteration of the protein preference. These results demonstrated that JTT-130 specifically decreased the fat preference. In our previous report, we demonstrated that the food suppressive effect of JTT-130 may be attributed to free fatty acids that have accumulated in the gastrointestinal tract [15]. Recently, several receptors that are activated by free fatty acids in the gastrointestinal tract have been identified in other studies, and these receptors reportedly play important roles in nutrition regulation [21, 22]. Furthermore, infusion of fatty acids into the intestine has been shown to reduce the food preference for fat [23]. Although further studies are needed to clarify the mechanism of action, it is possible that free fatty acids which have accumulated in the gastrointestinal tract as a result of the inhibition of fat absorption may contribute to the decrease in fat preference after treatment with JTT-130.

### 3.3. Effects of JTT-130 on Body Weight Gain

To evaluate the effect of JTT-130 on body weight due to total caloric intake, body weight was measured during the treatment period. JTT-130 significantly inhibited increases in body weight, and body weight gain observed during the treatment period was 50.7 ± 2.4 g in the control group and 37.4 ± 2.0 g in the JTT-130 group (*P* < 0.01). Interestingly, food efficiency during the treatment period also significantly decreased in the JTT-130 group (*P* < 0.05) compared with that of the control group (control group, 7.16 ± 0.21 g/100 kcal; JTT-130 group, 6.30 ± 0.27 g/100 kcal). These results indicate that inhibition of body weight gain by JTT-130 may be due to the previously reported increase in fecal excretion of fatty acids resulting from the inhibition of triglyceride (TG) absorption [12, 15], in addition to the decrease in total caloric intake.

### 3.4. Post-treatment Changes after Treatment with JTT-130

To evaluate whether the change in LF and HF diet intake observed during the treatment period with JTT-130 was reversed by discontinuation of JTT-130 treatment, LF and HF diet intake were measured for seven days following the cessation of treatment. During the seven-day recovery period, LF and HF diet intake and the ratio of LF and HF diet intake in the JTT-130 group tended to recover to a rate similar to what was observed in the control group (Figures 2 and 3(c)). In addition, total caloric intake in the JTT-130 group recovered to a similar level to what was observed in the control group (Figure 4(c)), and no significant differences were seen between the groups. These results suggest that the JTT-130-induced decrease in fat preference may be due to a decreased attractiveness of consuming fat rather than an avoidance response associated with taste aversion.

There were no differences in total caloric intake in the recovery period between the control and JTT-130 groups, showing that total caloric intake did not increase after discontinuation of treatment with JTT-130 (Figure 3(c)). When compared with a central anti-obesity agent such as sibutramine, which is a serotonin-noradrenaline reuptake inhibitor (SNRI) that is associated with a “rebound phenomenon” or increased food intake after discontinuation of treatment [24, 25], the same phenomenon was not observed with discontinuation of JTT-130 treatment.

### 3.5. Differences between JTT-130 and Existing Anti-Obesity Agents

In the present study, we have shown that JTT-130 specifically reduced the food preference for fat accompanied with a decrease in total caloric intake. As compared with existing anti-obesity agents in terms of the effect on food preference, sibutramine decreases both carbohydrate and fat consumption [26], showing that this agent has a different profile from that of JTT-130. Postmarketing surveillance studies of sibutramine showed that treatment with this agent increased cardiovascular risk, leading to a decision from the European Medicines Agency (EMA) and the US Food and Drug Administration (FDA) to withdraw the product from the market. Therefore, development of safer anti-obesity agents is necessary. Orlistat is known to reduce fat absorption by lipase inhibition and is used as an anti-obesity agent. However, orlistat does not decrease body weight as effectively as what is expected for the degree of fat malabsorption. This phenomenon is partly attributed to the compensation of energy loss due to the inhibition of fat absorption with increased energy intake [27]. In fact, we showed that orlistat increased cumulative food intake in high-fat diet-fed rats with suppressed food intake due to JTT-130 treatment [15]. Another report has also demonstrated that orlistat decreased fat preference. The authors proposed that orlistat might reduce the attractiveness of consuming fat due to fat malabsorption, although the mechanism remains unknown [28]. In contrast, JTT-130 specifically decreased fat preference without increasing total caloric intake. Moreover, orlistat has been shown to increase carbohydrate and protein consumption in a compensatory manner and, thus, also increase total caloric intake [28]. These findings indicate that JTT-130 and orlistat have different effects on energy intake and food preference, although both inhibit fat absorption locally in the gastrointestinal tract. After administration of orlistat, ingested fats may be found primarily in the form of TG in the intestinal lumen. After administration of JTT-130, in comparison, increases in TG and free fatty acids in the intestinal lumen and increases in TG in the small intestine tissue are observed [15]. These differences in types and amounts of ingested fats in the gastrointestinal tract may result in different effects of these compounds on food intake and food preference.

In conclusion, we have demonstrated that JTT-130, an intestine-specific MTP inhibitor, specifically decreases total caloric intake by reducing the preference for fat without changing the preference for carbohydrates, resulting in a reduction in body weight gain. After discontinuing JTT-130 treatment, total caloric intake returns to a similar level as the control and the reducing effect on the food preference for fat disappears. Thus, JTT-130 suppresses food intake with reducing the fat preference in addition to inhibiting fat absorption, suggesting that JTT-130 might be expected to provide a new option for the prevention and treatment of obesity and obesity-related metabolic disorders.

## Figures and Tables

**Table 1:** Composition of experimental diets.

Components	3.3% fat diet (LF diet)		35% fat diet (HF diet)	
	% (w/w)	kcal/kg	% (w/w)	kcal/kg
Soybean oil	2.5	225	2.5	225
Lard	0.8	72	32.5	2925
Corn starch	35.148	1406	3.448	138
Maltodextrin 10	12.5	500	12.5	500
Sucrose	15.0	600	15.0	600
Casein	24.0	960	24.0	960
L-Cystine	0.30	12	0.30	12
Cellulose oil, BW200	5.0	0	5.0	0
t-Butylhydroquinone	0.002	0	0.002	0
Mineral mix S10022M	3.5	0	3.5	0
Vitamin mix V10037	1.0	40	1.0	40
Choline bitartrate	0.25	0	0.25	0

Total	100.0	3815	100.0	5400

**Table 2:** Effect of JTT-130 on food intake from LF and HF diets during the experiment.

Study period	Baseline		Treatment		Recovery	
Groups	Control	JTT-130	Control	JTT-130	Control	JTT-130
	(g/day)					
LF diet	2.6 ± 0.5	2.4 ± 0.4	2.6 ± 0.6	6.9 ± 0.9	2.8 ± 0.5	6.3 ± 1.1
HF diet	16.5 ± 0.5	16.6 ± 0.6	16.7 ± 0.6	10.4 ± 0.6	17.6 ± 0.8	15.4 ± 0.8

Food intake from LF and HF diets was presented as average values during baseline, the treatment, and recovery period. Data are presented as means ± S.E. from ten animals.

**Table 3:** Effects of JTT-130 on daily macronutrient consumption during the treatment period.

	g/day		kcal/day	
	Control	JTT-130	Control	JTT-130
Carbohydrate	7.0 ± 0.4	7.7 ± 0.4	28.1 ± 1.5	30.8 ± 1.7
Fat	5.9 ± 0.2	3.9 ± 0.2**	53.4 ± 2.0	34.7 ± 1.6**
Protein	4.7 ± 0.2	4.2 ± 0.1*	18.8 ± 0.7	16.8 ± 0.5*

Macronutrient analysis was performed as described in Section 2. The caloric intake from each component was calculated using calories per unit weight of 4 kcal/g of carbohydrates and protein and 9 kcal/g of fat. *P < 0.05; **P < 0.01 versus control group.

## References

[1] Johnson PM, Kenny PJ (2010). Dopamine D2 receptors in addiction-like reward dysfunction and compulsive eating in obese rats. *Nature Neuroscience*.

[2] Welsh JA, Sharma A, Abramson JL, Vaccarino V, Gillespie C, Vos MB (2010). Caloric sweetener consumption and dyslipidemia among US adults. *Journal of the American Medical Association*.

[3] Greenwood CE, Winocur G (2005). High-fat diets, insulin resistance and declining cognitive function. *Neurobiology of Aging*.

[4] Takahashi M, Ikemoto S, Ezaki O (1999). Effect of the fat/carbohydrate ratio in the diet on obesity and oral glucose tolerance in C57BL/6J mice. *Journal of Nutritional Science and Vitaminology*.

[5] Drewnowski A, Brunzell JD, Sande K (1985). Sweet tooth reconsidered: taste responsiveness in human obesity. *Physiology and Behavior*.

[6] Mela DJ, Sacchetti DA (1991). Sensory preferences for fats: relationship with diet and body composition. *American Journal of Clinical Nutrition*.

[7] Mizushige T, Inoue K, Fushiki T (2007). Why is fat so tasty? Chemical reception of fatty acid on the tongue. *Journal of Nutritional Science and Vitaminology*.

[8] Gordon DA, Wetterau JR, Gregg RE (1995). Microsomal triglyceride transfer protein: a protein complex required for the assembly of lipoprotein particles. *Trends in Cell Biology*.

[9] Wetterau JR, Lin MC, Jamil H (1997). Microsomal triglyceride transfer protein. *Biochimica et Biophysica Acta*.

[10] Olofsson SO, Asp L, Borén J (1999). The assembly and secretion of apolipoprotein B-containing lipoproteins. *Current Opinion in Lipidology*.

[11] Xie Y, Newberry EP, Young SG (2006). Compensatory increase in hepatic lipogenesis in mice with conditional intestine-specific Mttp deficiency. *Journal of Biological Chemistry*.

[12] Mera Y, Odani N, Kawai T (2011). Pharmacological characterization of diethyl-2-({3-dimethylcarbamoyl-4-[(4′-trifluoromethylbiphenyl-2-carbonyl)amino]phenyl}acetyloxymethyl) -2-phenylmalonate (JTT-130), an intestine-specific inhibitor of microsomal triglyceride transfer protein. *Journal of Pharmacology and Experimental Therapeutics*.

[13] Hata T, Mera Y, Tadaki H (2011). JTT-130, a novel intestine-specific inhibitor of microsomal triglyceride transfer protein, suppresses high fat diet-induced obesity and glucose intolerance in Sprague-Dawley rats. *Diabetes, Obesity and Metabolism*.

[14] Hata T, Mera Y, Kawai T (2011). JTT-130, a novel intestine-specific inhibitor of microsomal triglyceride transfer protein, ameliorates impaired glucose and lipid metabolism in Zucker diabetic fatty rats. *Diabetes, Obesity and Metabolism*.

[15] Hata T, Mera Y, Ishii Y (2011). JTT-130, a novel intestine-specific inhibitor of microsomal triglyceride transfer protein, suppresses food intake and gastric emptying with the elevation of plasma peptide YY and glucagon-like peptide-1 in a dietary fat-dependent manner. *Journal of Pharmacology and Experimental Therapeutics*.

[16] Chaudhri O, Small C, Bloom S (2006). Gastrointestinal hormones regulating appetite. *Philosophical Transactions of the Royal Society B: Biological Sciences*.

[17] Murphy KG, Dhillo WS, Bloom SR (2006). Gut peptides in the regulation of food intake and energy homeostasis. *Endocrine Reviews*.

[18] Cummings DE, Overduin J (2007). Gastrointestinal regulation of food intake. *Journal of Clinical Investigation*.

[19] Warwick ZS, Schiffman SS (1992). Role of dietary fat in calorie intake and weight gain. *Neuroscience and Biobehavioral Reviews*.

[20] Sclafani A (2001). Psychobiology of food preferences. *International Journal of Obesity and Related Metabolic Disorders*.

[21] Hirasawa A, Hara T, Katsuma S, Adachi T, Tsujimoto G (2008). Free fatty acid receptors and drug discovery. *Biological and Pharmaceutical Bulletin*.

[22] Ichimura A, Hirasawa A, Hara T, Tsujimoto G (2009). Free fatty acid receptors act as nutrient sensors to regulate energy homeostasis. *Prostaglandins and Other Lipid Mediators*.

[23] Ogawa N, Ito M, Yamaguchi H (2012). Intestinal fatty acid infusion modulates food preference as well as calorie intake via the vagal nerve and midbrain-hypothalamic neural pathways in rats. *Metabolism*.

[24] Bray GA, Blackburn GL, Ferguson JM (1999). Sibutramine produces dose-related weight loss. *Obesity Research*.

[25] Fisas A, Codony X, Romero G (2006). Chronic 5-HT6 receptor modulation by E-6837 induces hypophagia and sustained weight loss in diet-induced obese rats. *British Journal of Pharmacology*.

[26] Leblanc M, Thibault L (2003). Effect of sibutramine on macronutrient selection in male and female rats. *Physiology and Behavior*.

[27] O’Donovan D, Feinle-Bisset C, Wishart J, Horowitz M (2003). Lipase inhibition attenuates the acute inhibitory effects of oral fat on food intake in healthy subjects. *British Journal of Nutrition*.

[28] Ackroff K, Sclafani A (1996). Effects of the lipase inhibitor orlistat on intake and preference for dietary fat in rats. *American Journal of Physiology*.

